# Practical application of microsphere samples for benchmarking a quantitative phase imaging system

**DOI:** 10.1002/cyto.a.24291

**Published:** 2020-12-20

**Authors:** Edward Kwee, Alexander Peterson, Michael Halter, John Elliott

**Affiliations:** Biosystems and Biomaterials Division, National Institute of Standards and Technology, Gaithersburg, Maryland

**Keywords:** HEK293, microspheres, quantitative phase imaging, reference materials

## Abstract

Quantitative phase imaging (QPI) provides an approach for monitoring the dry mass of individual cells by measuring the optical pathlength of visible light as it passes through cells. A distinct advantage of QPI is that the measurements result in optical path length quantities that are, in principle, instrument independent. Reference materials that induce a well-defined optical pathlength shift and are compatible with QPI imaging systems will be valuable in assuring the accuracy of such measurements on different instruments. In this study, we evaluate seven combinations of microspheres embedded in index refraction matching media as candidate reference materials for benchmarking the performance of a QPI system and as calibration standards for the optical pathlength measurement. Poly(methyl metharylate) microspheres and mineral oil were used to evaluate the range of illumination apertures, signal-to-noise ratios, and focus positions that allow an accurate quantitative optical pathlength measurement. The microsphere-based reference material can be used to verify settings on an instrument that are suitable for obtaining an accurate pathlength measurement from biological cells. The microsphere/media reference material is applied to QPI-based dry mass measurements of a population of HEK293 cells to benchmark and provide evidence that the QPI image data are accurate.

## INTRODUCTION

Microscopic evaluation of cells is fundamental in cell biology research and medical diagnostics. Due to the low difference in refractive index between cells and the surrounding cell culture media, visualization of unstained cells with brightfield imaging may not produce sufficient contrast for cell identification. Zernike phase contrast, which converts the phase change that is induced as light passes through cells to intensities visualized through a microscope, has been one of the more effective ways to visualize live unstained cells [1]. While useful for visualization, these intensities only serve as a qualitative measure of the phase change in light. Additionally, the Zernike phase contrast images are confounded by imaging artifacts, such as halos, that do not accurately represent the true phase change in the sample.

Quantitative phase imaging (QPI) is an imaging approach that quantitatively measures the change in phase of light passing through cells [2–5]. To ensure accurate and comparable QPI measurements from different instruments, the phase shifts can be made traceable to sharable reference materials. For example, reference materials in different formats have been used with QPI to demonstrate phase reconstruction capabilities. Custom planar reference materials have been developed using lithography techniques, some of which benefit from orthogonal characterization with atomic force microscopy, providing additional confidence in the dimensional features on the slide [6]. Although these reference materials provide confidence in instrument performance, they can be complicated to obtain in a typical biological laboratory.

To facilitate the in-house fabrication of reference materials for QPI, we evaluated the use of commercially available microspheres that are available with different indices of refraction. Microspheres have been widely used as reference materials for multiple QPI techniques [7–10]. Unlike other physical forms of other optical materials, microspheres can be used in well plates, a common format used in cell imaging. This would allow use of the microspheres alongside a cell culture to provide parallel measurement of the optical pathlength reference system over the course of an experiment. Microsphere refractive index can be characterized and verified using orthogonal optical methods such as surface plasma resonance imaging [11, 12]. One challenge with using these materials is that the optical pathlength change induced by polymeric microspheres is frequently quite different and not necessarily comparable to the pathlength change induced by biological cells.

In this study, we evaluated combinations of commercially available microspheres and immersion media with different refractive indices to identify combinations that result in optical pathlength changes that are similar to biological cells [13]. Immersion media studied included both liquid media and solid optical mounting media. Such combinations can become readily available resources as reference materials for the development and quality control of QPI technologies used for biological cell imaging. Such material can also be used to evaluate sources of variability in QPI imaging technologies. Here, we demonstrate the use of poly(methyl methacrylate) (PMMA) microspheres and mineral oil to evaluate the accuracy of QPI measurements with respect to varying illumination aperture, illumination energy, and focus position. QPI measurements of the microspheres in the mineral oil that deviated from the expected pathlength provided a clear indication of inappropriate instrumentation settings. These microsphere-based reference materials can be used to benchmark and verify settings on the instrument necessary for obtaining an accurate pathlength measurement from biological cells.

## METHODS

### Microsphere and liquid immersion media sample preparation

Optical pathlength variations were generated by immersing microspheres with an index of refraction into a liquid media with a different index of refraction. The liquid immersion media was selected to be either distilled water (*n* = 1.333), for direct comparison with aqueous cell media, or mineral oil (*n* = 1.468), for long-term stability (no evaporation) and attenuation of refractive index shift with high refractive index microsphere materials. Microspheres of various sizes and materials were obtained: Sephacryl S-300 microspheres (diameter 25-75 μm; GE Healthcare Biosciences, Pittsburgh, PA), polyacrylamide (PA) microspheres (Bio Gel P4, diameter < 45 μm; Bio-Rad, Hercules, CA), PMMA microspheres (diameter 63-75 μm; Cospheric, Santa Barbara, CA), polystyrene (PS) microspheres (6 μm, 10 μm; Thermo Fisher Scientific, Waltham, MA), and silica microspheres (diameter 6.1 μm; Bangs Laboratories, Inc., Fishers, IN). If shipped as a suspension, 100 μl of stock microsphere suspension was diluted at a 1:10 ratio in distilled water and vortex mixed for 60 s. This dilution was centrifuged and then resuspended with 1 ml ultrapure distilled water, repeated twice. Silica and PMMA microspheres were shipped dry and resuspended in a 10:1 dilution by volume in a microfuge tube using ultrapure distilled water and vortex mixed for 60 s. Imaging was performed on either a glass microscope slide or in a 12-well multiwell plate. For glass slide imaging, 10-20 μl of the water-microsphere suspension was added to a microscope slide before being covered with a glass coverslip. For multiwell plate imaging, 100 μl of the water-microsphere suspension was added to one well of a 12-well plate. An additional 1 ml ultrapure distilled water was added to the well to achieve a single microsphere suspension. The microspheres were allowed to settle onto the surface before imaging. For study of microspheres suspended in oil, the silica, PMMA and PS microspheres were suspended in 1 ml mineral oil (BioUltra, Sigma-Aldrich, St. Louis, MO). This mineral oil product was labeled by the manufacturer with lot-to-lot characterization of the refractive index. For multiwell plate imaging, 100 μl of the mineral oil-microsphere suspension was added to one well of a 12-well plate with an additional 1 ml of mineral oil.

### Microsphere and solid immersion media sample preparation

Solid optical mounting media to permanently mount the microsphere materials onto the slide were chosen based on having the appropriate refractive index difference between the microspheres and mounting media. Glass microscope slides and coverslips were acid cleaned and used to mount microspheres of various sizes and index of refraction with solid optical mounting media Meltmount (*n* = 1.539; *n* = 1.582; Cargille Laboratories, Cedar Grove, NJ) according to the manufacturer’s protocol. Briefly, a 10-20 μl droplet of the water-microsphere suspension was added to a glass coverslip and allowed to air dry overnight. The glass coverslip and microscope slide were placed on a hot plate and heated to 65°C. The Meltmount in Quick-Stick rod was placed in contact with the heated microscope slide to transfer the mounting media. The coverslip with dried microspheres was placed over the mounting media on the microscope slide and allowed to cool to room temperature creating a permanently mounted microscope slide.

### QPI acquisition

QPI was performed using a quadriwave lateral shearing interferometer (SID4-Bio, Phasics, France) [7] directly attached to the camera port of an Axiovert 200 M inverted microscope (Carl Zeiss Microscopy, Thornwood, NY) with motorized condenser (Part No. 1005-848). The initial evaluation of microsphere and mounting media combinations was performed using an incoherent white light source (HAL 100; Carl Zeiss Microscopy) configured for Kohler illumination through a 590 nm notch filter (Part No. FB590-10; Thor Labs, Newton, NJ). Subsequent evaluation of sensitivity of QPI acquisition settings and cells was performed using a 590 nm centered LED (Part No. M590L3-C4; Thor Labs) for illumination. Images were collected using either a 10X Plan-Apochromat 0.45 NA air objective (Part No. 420640-9900-000; Carl Zeiss Microscopy) or 40X 0.75 NA air objective (Part No. 440350-9903-000; Carl Zeiss Microscopy, Thornwood, New York). Images were acquired using the SID4-Bio acquisition software driving Micro-Manager via a plugin interface [14]. A reference image of the background media, required for this QPI imaging system, was also acquired. The subsequent sample images were recorded as optical pathlength difference (*OPD*) in nanometer units relative to the background sample.

### QPI analysis of microsphere combinations

The phase shift images of microsphere and immersion medium combinations were exported from the SID4-Bio acquisition software as 32-bit TIFF files and imported into FIJI image analysis software [15]. The spatial pixel calibration of the image was validated with a linear spatial calibration target (Cat No. 68042-08; Electron Microscopy Services, Hatfield, PA). Microsphere segmentation was performed using the default threshold algorithm, based on the IsoData algorithm [16], in FIJI for evaluation of the images of the microsphere and immersion media preparations. The remaining background pixel intensities were averaged and subtracted uniformly across all pixels in the image to correct the *OPD* image for offsets that can result from differences between the reference and sample images [7].

The maximum *OPD* signal was determined for each segmented object. The object diameter was determined from the corresponding brightfield image. For each set of images (*n* ≥ 3) corresponding to each microsphere-immersion media combination, the range of maximum *OPD* values for each microsphere were complied. For each microsphere object, the measured diameter (*D*) was used to calculate the change in refractive index (Δ*n*) by dividing the maximum measured *OPD* value according to Δ*n* = OPD/D. *OPD* value calibration was validated by comparing a microsphere/immersion media combination with the known microsphere refractive index values measured by surface plasmon resonance imaging [17]. The analysis was repeated for all microsphere and immersion medium combinations. The measured Δ*n* value between the microsphere and the immersion media was within 5% of the expected value for all microsphere and immersion media combinations, with correct focus and illumination settings.

For the combination of PMMA microspheres and mineral oil, two lots of PMMA microspheres and three lots of mineral oil were assessed for refractive index and Δ*n* to analyze preparation variability using different microsphere and mineral oil lots.

### Sensitivity of QPI-derived OPD to illumination aperture, illumination energy, and focus

PMMA microspheres in mineral oil were used to assess the sensitivity of QPI measurements to changes in acquisition parameters. Baseline acquisition settings were set with the condenser numerical aperture (NA) at the smallest possible setting of the microscope condenser aperture at 0.09, illumination fluence of 0.19 μJ/mm^2^ (1.9 μW/mm^2^ illumination irradiance × 100 ms exposure time) and acquired at the in focus focal plane determined by manual inspection. When the condenser aperture size was increased, the exposure time was adjusted so that the mean image intensity remained constant at approximately 3000 intensity units. Illumination energy was varied above and below the baseline settings while maintaining a constant exposure time. Under all aperture setting, exposure time, and focal plane conditions, a reference image was acquired using a background sample identical to the corresponding PMMA microspheres in mineral oil sample except without microspheres. The appropriate reference image was used in the computation of the phase image. The active contour method was implemented in MATLAB 2017b (Mathworks, Natick, MA) to segment microspheres in the phase image. The contraction bias setting of the active contour method was manually adjusted for appropriate bead edge detection. This parameter was used to segment all images collected for the microscope sensitivity analysis. The PMMA microsphere refractive index was measured as described above. Microsphere optical volume was calculated by summing the optical pathlength difference within the segmented microsphere area. Percent error of microsphere refractive index measurements was calculated with respect to the microsphere refractive index measured under the baseline acquisition settings. Percent error of microsphere optical volume was calculated with respect to theoretical optical volume with an average microsphere diameter of 67.7 μm.

### QPI of live HEK293 cells

Time lapse images were acquired from live HEK293 cells. HEK293 cells were cultured in EMEM media imaged using QPI every 15 min for 2 days. A stage top incubator (Kairos Inc., Pittsburgh, PA) was used to maintain humidified cell culture conditions at 37°C and 5% CO_2_. HEK293 cells were imaged under a similar range of illumination aperture, illumination energy, and focus conditions as the PMMA microspheres. Image analysis of the time lapse image sets was performed by segmenting cells using the empirical gradient threshold method implemented in MATLAB, which performs automated segmentation based on image gradient thresholding [18]. Cell optical volume for the entire field of view was calculated by summing the *OPDs* within the segmented area. Cell mass was determined from the optical volume using a specific refractive increment of 1.8 × 10^−4^ m^3^/kg, [3, 19] estimated from cellular components. Cell mass density was calculated by dividing the cell mass by the segmented area. Percent change of the cell mass, area, and density were calculated with respect to the baseline acquisition parameters, as described in the PMMA microsphere imaging method.

## RESULTS

### Survey of candidate microspheres

Five microsphere materials with a range of refractive indices and diameters were evaluated across seven immersion materials, both liquid and solid, with a range of refractive index values. The expected difference in Δ*n* and *OPD* was calculated based on measurements reported in literature or based on product specifications provided by the manufacturer and adapted to λ = 590 nm (Table S1). From these microsphere/media combinations, we applied the following criteria for selection as a candidate reference material combination for biologic samples: (1) A refractive index difference (Δ*n* < 0.05) that is similar to the difference between cells and culture media. (2) An *OPD* that is within the range of either adherent or nonadherent mammalian cells. (3) Microspheres appeared with well-defined edges when observed using brightfield microscopy so that the diameter of individual microspheres can be unambiguously determined. The microsphere/media combinations were also evaluated qualitatively based on eight fit-for-purpose attributes, including fabrication ease and stability. A comparison of the microsphere preparations is summarized in Table S2.

**FIGURE 1:**
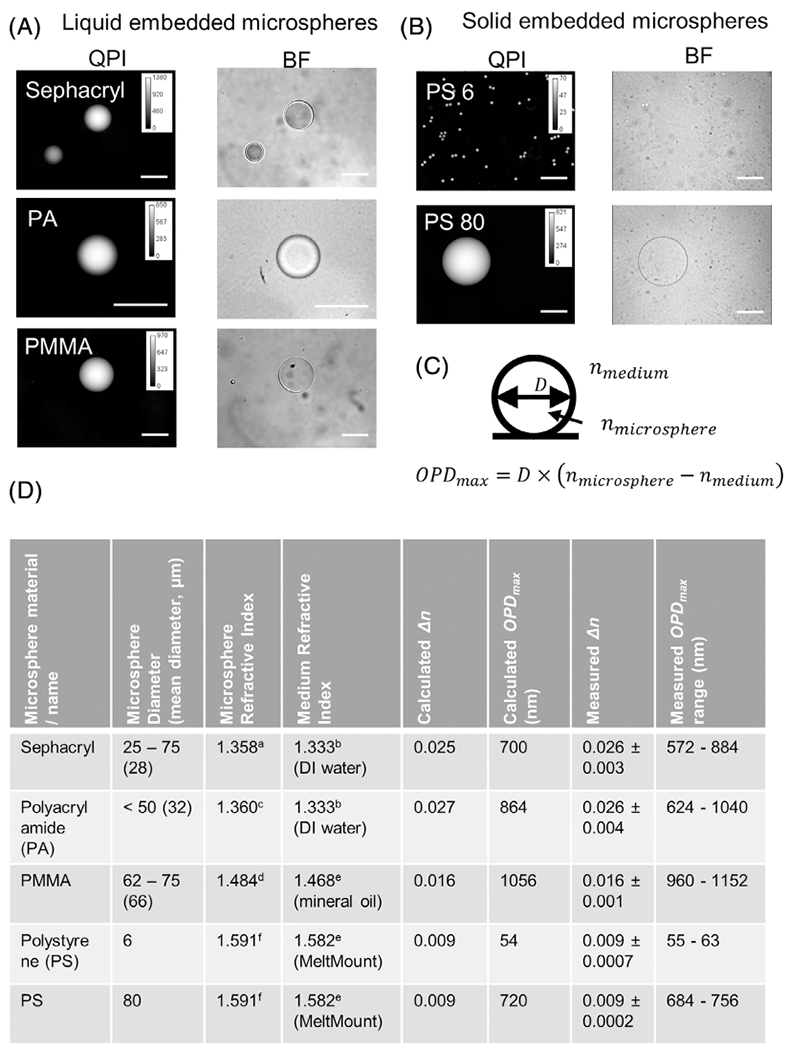
Evaluation of microspheres embedded into liquid or solid immersion media using quantitative phase imaging (QPI). (A) QPI and bright field (BF) images of candidate microspheres dispersed into liquid immersion media. (B) QPI and BF images of polystyrene microspheres embedded into solid immersion media. (C) The measured Δ*n*, which is nmicrosphere-nmedium, was determined by dividing the microsphere diameter, D, by the maximum OPD, OPDmax, of the microsphere. The average Δ*n* (± standard deviation) value for each microsphere/media combination was compiled for a minimum of 20 different microspheres. D was determined from the brightfield image and OPDmax was determined from the QPI image (see Methods section for analysis description). (D) Table of microsphere parameters both calculated and measured (λ = 590 nm) that show close agreement for change in refractive index (Δ*n*) and optical phase difference (OPD). Scale bar for images in (A) and (B) is 50 μm. Phase shift shown in nm

Five microsphere/media combinations met the selection criteria and were further characterized using dual-mode imaging and an image analysis pipeline. Brightfield images were used to determine the diameters of individual microspheres and QPI images were used to quantify the *OPD* induced by the microspheres as shown in Figure 1. QPI shows high contrast images of *OPD* while brightfield imaging shows low contrast for both microsphere/liquid media combinations (Figure 1(A)) and microsphere/solid media combinations (Figure 1(B)).

After microsphere segmentation with the brightfield image, the maximum *OPD* value at the center of the microsphere was obtained from the corresponding *QPI* image with image analysis. The change in the refractive index is determined by the relationship *OPD* = Δn*D, where *D* is the microsphere diameter (Figure 1(C)). The measured Δ*n* values are compared alongside the expected Δ*n* values based on the refractive indices of the immersion media and the microspheres in Figure 1(D) [11, 17, 20, 21]. The values of *OPD* range are shown to indicate the overall expected variability in the *OPD* magnitudes that arises primarily because of the dispersion in microsphere diameters.

For PMMA and mineral oil, six preparations of reference material were made in combination with the three mineral oil lots and two PMMA microsphere lots and analyzed. Using the baseline QPI acquisition parameters, QPI measurements were taken across multiple images (*n* > 7) and microspheres (*n* > 52). Mineral oil refractive index varied (Table S3) and the resulting Δ*n* varied, ranging between 0.0154 and 0.0220 (Table S4). The variability of the Δ*n* and resulting PMMA refractive index measurement between five independent preparations of the same lot of microspheres and mineral oil was 0.00042 (Table S5). Additionally, the measured refractive index of the two lots the PMMA microspheres was 1.486 and 1.490, respectively.

### Evaluation of QPI-derived OPD measurement to changes in illumination aperture, illumination energy, and focal plane

**FIGURE 2:**
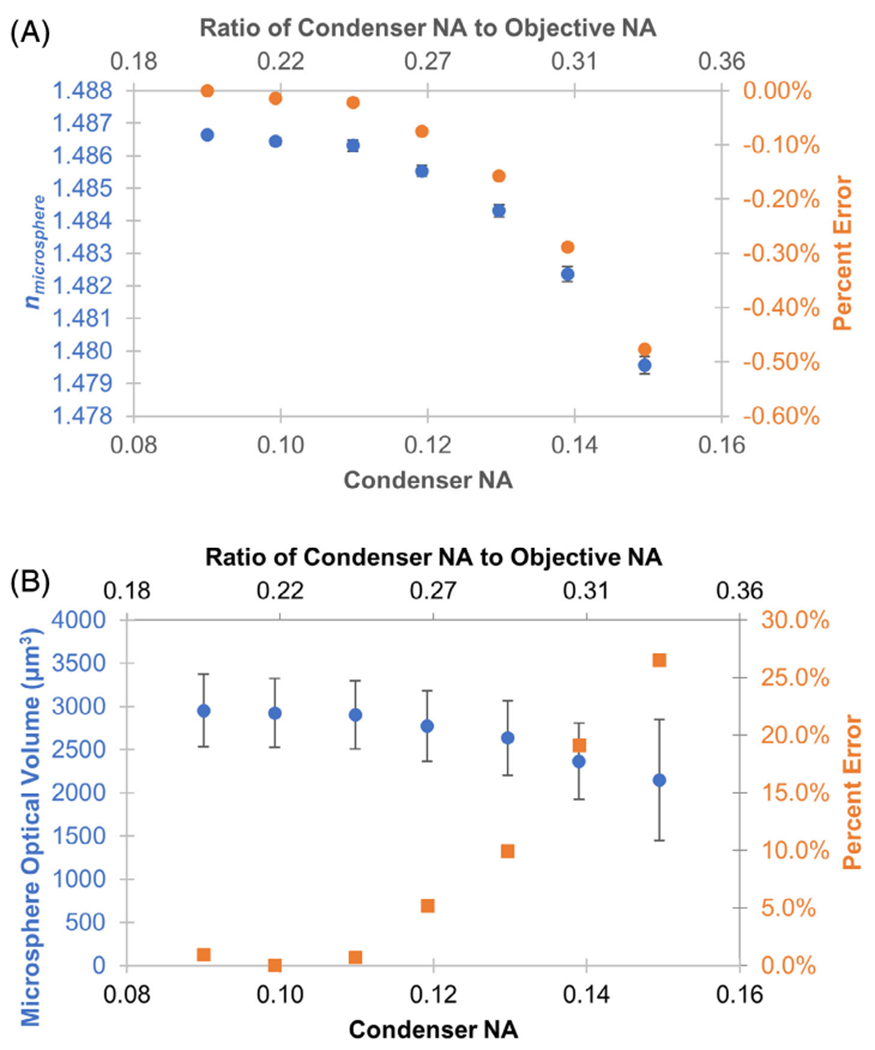
Evaluation of microsphere refractive index (A) and optical volume (B) measured by QPI in response to varying illumination aperture. Microsphere phase image reconstruction was not possible at aperture settings above 0.15. Error bars represent standard error of the mean across 66 microspheres in the image. Percent error of refractive index and optical volume were calculated with respect to the optimal condenser N.A. setting at 0.09 and average microsphere diameter of 67.7 μm

The PMMA microsphere and mineral oil immersion medium combination (Figure 1) were used to measure the measurement sensitivity to changes in illumination aperture, illumination energy, and focal plane. This immersion media and microsphere combination were stable over several weeks (Table S2). Representative images of PMMA microspheres in mineral oil under varying acquisition settings are shown in Figure S1. The importance of the ratio of the illumination numerical aperture (NA) to the microscope objective NA has been highlighted when using the transport of intensity QPI approach [22]. The ratio is critical because it provides a benchmark measure of the relative coherence of the illumination light. These reference materials and protocols can also be used to establish that the illumination NA for a given optical configuration is appropriate for QPI. Measurement of this ratio for an imaging system will provide supporting evidence that the phase information recorded in the image is accurate. NA settings less than 0.12 resulted in accurate optical volume measurements with less than 5% error and refractive index measurements with less than 0.10% error (Figure 2). This corresponded to an illumination NA to objective NA ratio of 0.26.

**FIGURE 3:**
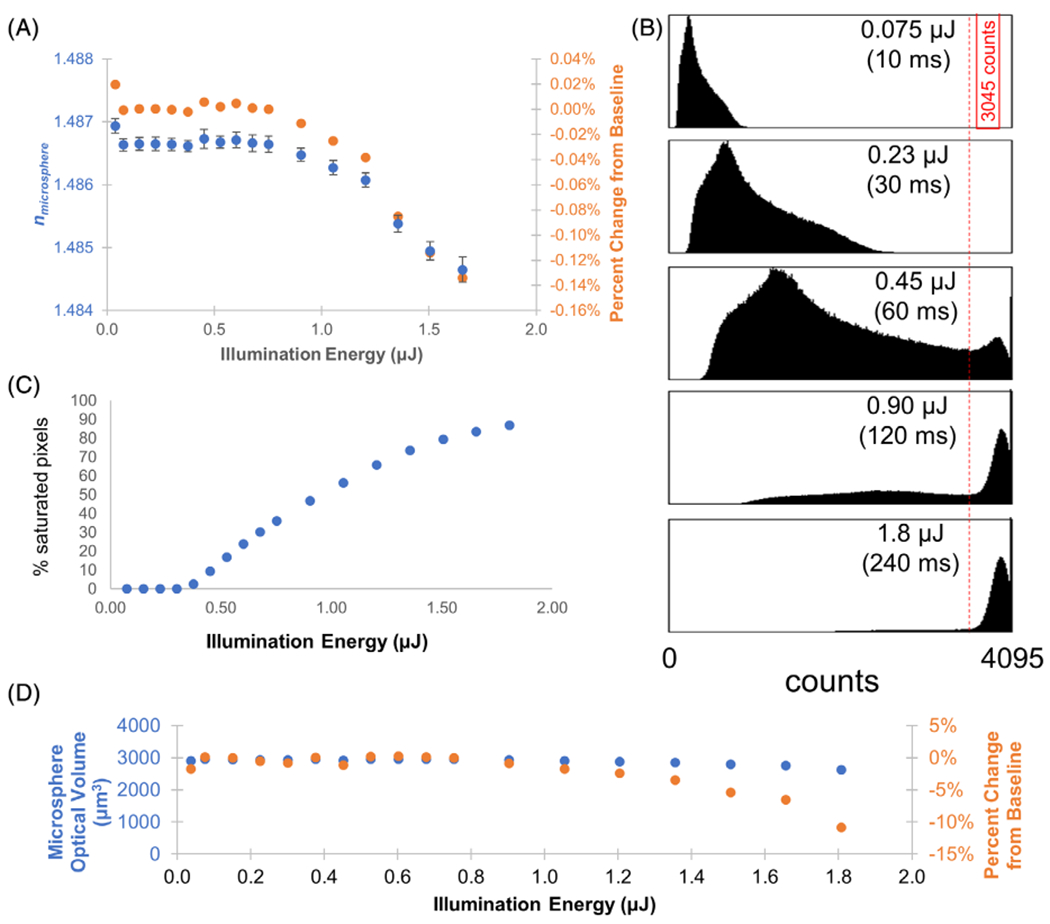
Evaluation of the measured Δ*n* and microsphere optical volume in response to illumination energy. The sample illumination energy produced by a LED illumination source centered at 590 nm was varied by changing the detector exposure time. The measured illumination power at the specimen plane was 7.5 μW over an area of 3.86 mm^2^. (A) Plot of the mean Δn for 66 beads versus the illumination energy. Error bars represent standard error of the mean. (B) Histogram of the CCD intensities (counts) for five different light exposure levels. Pixel saturation is indicated by the accumulation of pixel counts above the red vertical line. (C) A threshold at 3545 counts (red vertical line) was selected to quantify the number of saturated pixels. The mean on deviates from the baseline value when approximately 50% of the pixels are saturated. (D) Percent error of refractive index and optical volume were calculated with respect to the optimal illumination energy of 0.8 μJ and average microsphere diameter of 67.7 μm

The sensitivity of the measured Δ*n* induced by PMMA microspheres in mineral oil upon changing the signal at the array detector was evaluated in Figure 3(A). The signal was varied by changing the exposure time, which resulted in a corresponding change in the sample illumination energy. Over a range of illumination energies from 35 nJ (nanojoule) to 1 μJ (microjoule), the measured Δ*n* was approximately constant. Accurate Δ*n* estimates were obtained at the lowest light dosage examined, 35 nJ. The only deviation of the measured Δ*n* from the expected value corresponded to saturation of the CCD array detector, illustrated with the intensity histogram plots in Figure 3(B). At an illumination energy of 1 μJ, the plot of illumination energy versus percent saturated pixels in Figure 3(C) shows that approximately 50% of the detector pixels were saturated (i.e., pixel responses were nonlinear with increasing light levels). The corresponding change in microsphere optical volume estimates with illumination energy is shown in Figure 3(D). While the experiments in this study do not explore low light levels at which the Δ*n* and optical volume measurements are degraded, an analysis of the detector noise [23] is presented in Text S1, which indicates an exposure time of 135 μs would be sufficient to achieve a pixel signal to noise of approximately 3. Using the illumination conditions in Figure 3, illumination power of 7.5 μW over an area of 3.86 mm^2^, the corresponding illumination energy is 0.001 μJ. It is expected that Δ*n* and optical volume measurements would become significantly degraded at these low light levels.

**FIGURE 4:**
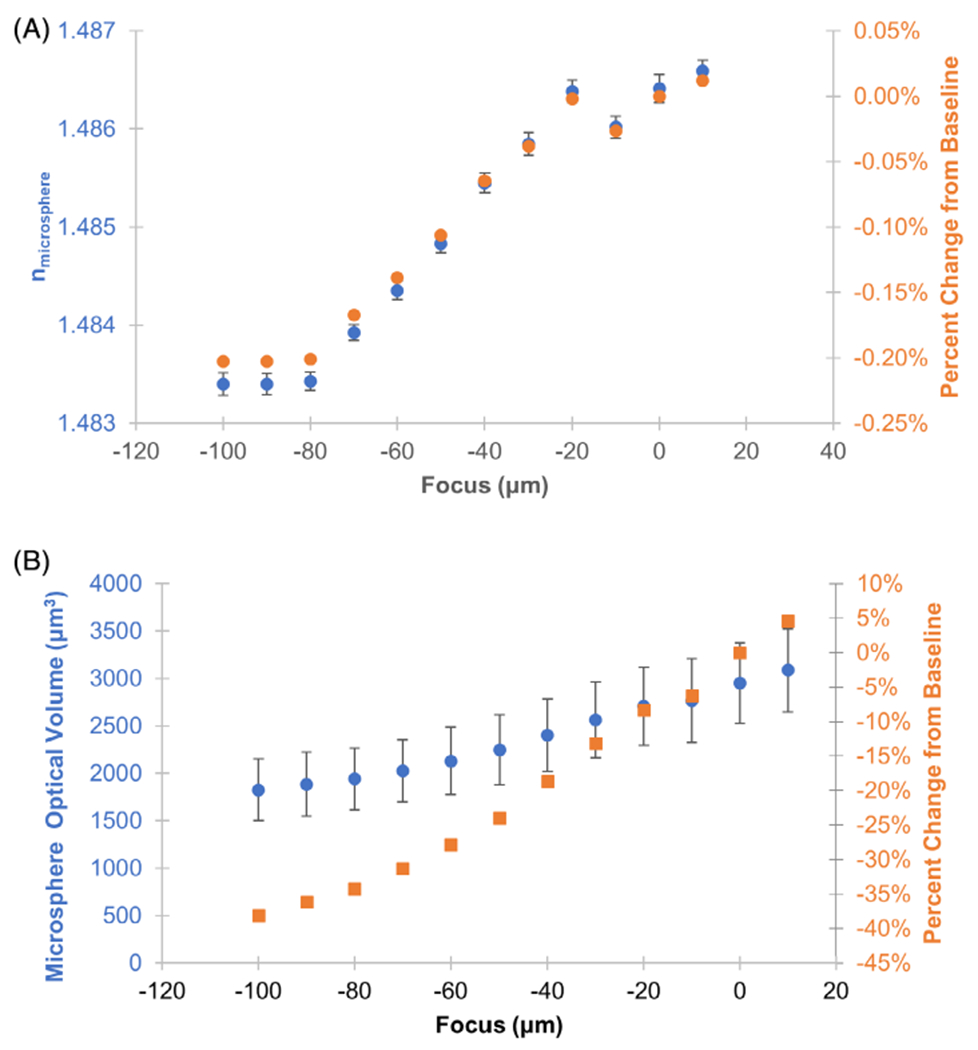
Evaluation of quantitative QPI results in response to varying image focus. (A) Plot of the estimated index of refraction of the PMMA microsphere material versus sample focus. (B) Plot of the estimated microsphere optical volume versus sample focus. The in-focus image plane was determined by inspection and labeled 0 μm focus. Microsphere phase image reconstruction was not possible for focus values above 10 μm. Error bars represent the standard error of the mean across 66 microspheres. Percent error of refractive index and optical volume were calculated with respect to in focus plane at 0 μm and average microsphere diameter of 67.7 μm

Sample focus is a well-known factor that can strongly influence quantitative imaging results [24–26]. The sensitivity of *OPD* and optical volume to sample focus was evaluated using PMMA microspheres in mineral oil. Focus within −30 to 10 μm from the in-focus plane resulted in measurements with less than 0.05% error in microsphere refractive index and less than 15% error in microsphere optical volume (Figure 4). The measured microsphere refractive index decreased at focus positions below −30 μm from the in-focus plane, but only reached a 0.23% error at −100 μm. The microsphere optical volume and diameter measurements also decreases below −30 μm from the in-focus plane, with the error reaching 40% at −100 μm.

In total, these analyses demonstrate the application of the PMMA microspheres and mineral oil immersion medium as a reference material for establishing the illumination energy, illumination condenser aperture, and focus ranges over which a QPI system will provide accurate measures of *OPD*.

### Evaluation of HEK293 mass measurements to changes in illumination aperture, illumination energy, and focal plan

**FIGURE 5:**
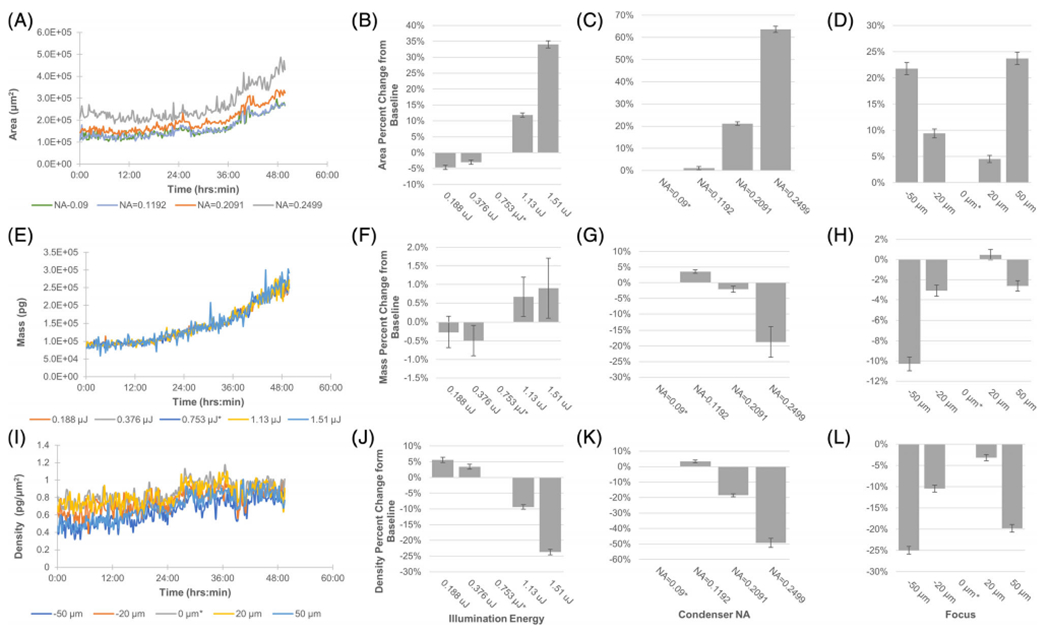
Time lapse imaging of HEK293 under varying QPI acquisition parameters for illumination energy, condenser NA, and focal plane. Analysis performed on 10x field of view. (A) Dynamic cell area measurements with varying condenser NA. (B-D) Area percent change compared to baseline setting. (E) Dynamic cell mass measurements with varying illumination energy. (F-H) Mass percent change compared to baseline setting. I) Dynamic mass density measurements with varying focus. (J-L) Density percent change compared to baseline setting. Standard error of the mean shown in error bars (*n* = 200 time points). Baseline acquisition condition identified by asterisk (*)

Images were acquired of HEK293 cells for acquisition parameters corresponding to those taken with PMMA microspheres in mineral oil (Figure 5). Representative cell images are shown in Figure S2. Nominal measurements were generated from images collected under baseline acquisition parameters (see Methods Section). The resulting cell mass and area measurements were also dependent on the microscope acquisition settings. Cell area, mass, and density measurements remained within 5% of the nominal values when the illumination energy was less than 0.8 μJ and the condenser illumination aperture was less than 0.12. These measurements varied less than 10% from their nominal values when focus was within −20 μm and 20 μm of the in-focus plane. These ranges of acquisition parameters are highly similar to the ranges identified for accurate microsphere measurements.

The automated segmentation method generated different cell segmentation masks under the different acquisition settings. High condenser NA settings resulted in the highest increase in cell area when compared to that obtained under baseline acquisition conditions (Figure 5(A,C)). In the case of illumination energy, while the cell area did vary up to 34% based on illumination energy (Figure 5(B)), the resulting cell mass measurements changed less than 1% when compared to baseline (Figure 5(F)). This is due to the additional segmented area representing mainly background pixels that did not contribute to the measured cell mass. Mass density measures with varying focus showed high changes from nominal values due to sensitivity to changes in both mass and area.

## DISCUSSION

Reference materials can be applied in different ways to characterize an analytical measurement system [27]. Often, they are used to validate instrument calibration, such as using a material with a known *OPD* to ensure a QPI measurement system is calibrated. Another application of reference materials is analytical performance benchmarking to assure that an instrument is operationally capable of performing a specified measurement [23, 28]. Easily accessible reference materials, such as those described here, would facilitate the interpretation of results derived from QPI by providing assurance the quantitative results are reliable and comparable. However, it is important that the reference materials are fit-for-purpose for cellular measurements. Identifying the microsphere and media combinations that provide an optical pathlength shift similar to biological cells is challenging. Most well-characterized microsphere materials have a refractive index value that is much larger than that of biological cells (*n* ≈ 1.38) and cell culture media (*n* ≈ 1.33). The appropriate attenuation of the *OPD* induced by microspheres requires screening multiple available microsphere/immersion media combinations for samples that provide an *OPD* similar to biological cells, are chemically stable, and have a form factor and size range that is useful for optical microscopy. Overall, the measured values of Δ*n* for each microsphere/immersion media combinations are in close agreement with the calculated values. The measured OPD for 6 μm PS microsphere in MeltMount deviated from expected values by 1 nm. Calibration of our QPI system was validated with known Δ*n* values for microsphere/media combinations determined by orthogonal measurements (see Methods section). Most measured microsphere/media *OPD* values more closely match *OPD* values that would be expected for nonadherent or loosely adherent round cells (≈900 nm phase shift, see Figure S2). We did not identify any microsphere/media combination with *OPD* values that would be consistent with adherent cells (≈500 nm phase shift). It is possible to achieve lower *OPD* values by using microspheres less than 10 μm in diameter, but in our experience, the increased relative uncertainty in the estimated microsphere diameter, due to the system resolution and segmentation errors, caused an increased uncertainty in the *OPD* value. Regarding microsphere diameter measurement for calculating Δ*n*, we found measurements by brightfield transmitted microscopy differed from measurement by QPI to be less than 1% (see Figure S3), suggesting both can be used to determine a consistent diameter

Table S2 provides criteria that can be used for selecting microsphere/media combinations for specific applications of QPI where the cell sample under study and the particular QPI implementation can vary. Attributes such as specific physical formats, low-cost materials, and simplicity in fabrication and usage are evaluated for possible real-world reference material design and utility. PS microspheres solid embedded in Meltmount on a glass slide can serve as a durable reference material. PMMA microspheres used in mineral oil is easy to place in the bottom of a multiwell tissue culture plate and is stable over several weeks. We also highlighted five microsphere/media combinations that met our index of refraction (Δ*n* < 0.05), *OPD* criteria for cells (phase shift less than 1500 nm), and desirable fit-for-purpose qualities (indicated with yellow in Table S1).

QPI approaches that utilize a traditional microscope, like quadriwave lateral shearing and transport of intensity equation, have a wider range of acquisition parameters that can be adjusted than other self-contained QPI instruments. Incorrect microscope settings can significantly affect the accuracy of QPI measurements but have not been thoroughly evaluated [22]. To assess the utility of microspheres as a benchmarking material, phase shift images of the PMMA microspheres in mineral oil were acquired under a range of conditions. Different microsphere and mineral oil lots have different refractive indices. Because of this variation, reference material preparations using different lots of microspheres and mineral oil result in different values of Δ*n*. A single lot of microspheres and mineral oil can improve consistency in Δ*n* and may be more useful as a reference material for day to day QPI instrument calibration or instrument to instrument comparability assessment.

The quadriwave lateral shearing approach to determining the QPI relies on computational processing of the signal captured by the image sensor (CCD) to reconstruct a derived image representing the phase retardation of light as it passes through the sample. While it is possible to predict the CCD response to changes in incident light levels (illumination energy) [23, 29], the QPI algorithm response can be more complicated. The evaluation of QPI acquisition parameters identified conditions which produced low error in PMMA microsphere refractive index and optical volume measurements. For condenser NA, a range of values were tested between 0.08 and 0.16. This corresponded to evaluated illumination NA to objective NA ratios of 0.18-0.36. Previous measurements of optical volume sensitivity using the quadriwave lateral shearing approach showed low error for illumination NA to objective NA ratios less than 0.3 [30]. For illumination NA to objective NA ratios greater than 0.3, microsphere optical volume measurement error exceeded 19%. Ratios less than 0.3 resulted in less than 10% optical volume measurement error. Illumination to objective NA ratios less than 0.3 is necessary to impose spatial coherence in the illuminating light source, a requirement for the quadriwave lateral shearing approach of QPI [7]. This requirement also closely corresponds to the same required ratio of 0.3 for the transport of intensity approach to QPI [22]. Illumination intensity less than 0.8 μJ and focus between −30 and +10 μm from the in-focus plane were necessary to construct a phase image to accurately reproduce microsphere refractive index and optical volume measurements. HEK293 cell measurements were consistent with microspheres with illumination aperture of less than 0.12 and illumination energy less than 0.8 μJ resulting in less than 5% change in cell area, mass, and density compared with nominal values. HEK293 cell images could be produced at focus above 10 μm and resulted in less than 10% change in cell measurements up to 20 μm from the in-focus plane. This difference could be explained by the smaller *OPD* gradient in the cells compared to the microspheres. Because the *OPD* gradient of image can be reduced at higher magnifications, the focus range is magnification dependent and the sensitivity should be re-evaluated at different magnifications.

For time lapse QPI measurements of HEK293 cells, cell area was the most sensitive image analysis feature to changes in microscope parameters (condenser NA, illumination energy, focus) that can result in errors of more than 60% cellular area when parameters were extremely off and 10% error in cell area with moderately off baseline settings. Cell density values, relying on area measurements, were consequently just as adversely affected. Here, a single segmentation algorithm was used to replicate a workflow where a set algorithm was used to analyze cell area in a study. The errors in cell area could be compensated by retrospectively identifying new image analysis parameters for each different acquisition setting. The reference material can aid in identifying when image analysis modifications are needed. However, as image quality degrades with out of specification acquisition parameters (Supplemental Figure 2), the accuracy of resulting cell area measurements, even with modified analysis parameters, cannot be ensured. The reference material can be used to ensure correct acquisition settings and accurate cell area measurements. In contrast, cell mass measurements were the least sensitive to unoptimized microscope parameter settings, varying from minor 2% error with moderately poor settings to greater than 10% errors in cell mass at off parameter settings. Overall, the image acquisition parameters identified for accurate microsphere measurements corresponded to acquisition parameters for accurate HEK293 measurements. This suggests that the microsphere/media combinations have optical and scale properties similar to biological cells which can be ideal for a reference material.

Microspheres immersed in an appropriate medium may serve as a reference material to enable accurate QPI measurements of optical pathlength. The measurements described here were collected with a quadriwave lateral shearing QPI instrument, but it is likely that the microsphere reference materials will be useful in other QPI techniques such as transport of intensity [31], holography [13, 32, 33], and ptychography [6]. Future interlaboratory and inter-instrument studies would be valuable to further demonstrate the utility of reference materials to benchmark and enable comparability between QPI measurements. We use these reference materials to identify the range of acquisition conditions that produce unbiased quantitative phase measurements. This benchmarking strategy can provide assurance that an instrument is generating accurate phase information during an experiment.

## Supplementary Material

Supplemental Text 1

Supplemental Figure 1

Supplemental Figure 3

Supplemental Table 1

Supplemental Figure 2

Supplemental Table 2

Supplemental Table 3

Supplemental Table 4

Supplemental Table 5
