# Supplementary material for: Practical application of microsphere samples for benchmarking a quantitative phase imaging system
Source: Cytometry A. Author manuscript; Available in PMC 2022 Oct 1. (PMC8195315; doi:10.1002/cyto.a.24291)
Supplement: Supplemental Text 1 [file NIHMS1701327-supplement-Supplemental_Text_1.docx]

Supplemental Text 1

In this section we consider the QPI measurement of microsphere *Δn* and optical volume measurements under low light conditions. The experimental data in this study do not explore low light level conditions. However, the data acquired in this study can be used to compute signal to noise ratio trends that can be used to anticipate signal degradation at low light levels. The following analysis is based on a pixel level analysis, speculating that accuracy in pixel level intensity measurements is required for accurate phase retrieval from the raw interferograms.

As a first step, the Phasics camera used in this study was benchmarked according to Halter et al. [23] Briefly, a series of image pairs was acquired over a range of exposure times. The images are used to compute the mean pixel intensity, $I$, and the pixel variance, which vary as a function the exposure time. Plotting the pixel variance versus the mean pixel intensity, $I$, as in Supplemental Text, Figure 1 can be used identify the linear range of the detector and the expected variance for a given mean pixel intensity, $I$. The signal to noise ratio, $SNR,$ for a given mean pixel intensity, $I$, is computed as

$SNR=\frac{I}{\sqrt{\left( 0.1121\times I \right)+5.6604}}$ (1),

where the denominator is the square root of the pixel variance computed from regression analysis of the pixel variance versus the mean pixel intensity, $I$, shown in Supplemental Text 1, Figure 1.

Supplement Text 1, Figure 1: Plot of the pixel variance in each frame versus mean intensity. Image pairs were acquired at increasing exposure times while the microscope condenser maintain a constant illumination. As the pixel intensity increases, the pixel variance increases at first, reaches a maximum, then decreases. Within the linear range of the detector, the pixel variance varies proportionally with the pixel intensity. The data within the linear range were fit by linear regression (solid line) with the fit parameter displayed on the plot. Instead of using stably fluorescent glass under fluorescent imaging conditions as in Halter et al., the condenser lens of the microscope under Kohler illumination conditions was used as a stable light source.

The mean pixel intensity, $I$, is plotted versus $SNR$ in Supplemental Text 1, Figure 2 and indicates that $I=28$ corresponds to a pixel $SNR$ of approximately 3. Regression analysis of the illumination energy versus $I$ plot in Supplemental Text 1, Figure 3 can be used to compute that an illumination energy of 0.001 µJ corresponding to $I=28$ . For the illumination power used in this study of 7.5 µW over an area of 3.86 mm^2^, an exposure time of 135 µs would be sufficient to achieve a pixel$SNR$ of approximately 3.

Supplemental Text 1, Figure 2. Plot of $SNR$versus mean pixel intensity, $I$.

Supplemental Text 1, Figure 3. Plot of illumination energy (mJoules) versus mean pixel intensity, $I$. The illumination energy was computed for different exposure times from the illumination power. The illumination power from the transmitted light condenser under Kohler illumination conditions was measured using a power meter (see Methods).
