# Supplementary figures and images for: Practical application of microsphere samples for benchmarking a quantitative phase imaging system
Source: Cytometry A. Author manuscript; Available in PMC 2022 Oct 1. (PMC8195315; doi:10.1002/cyto.a.24291)

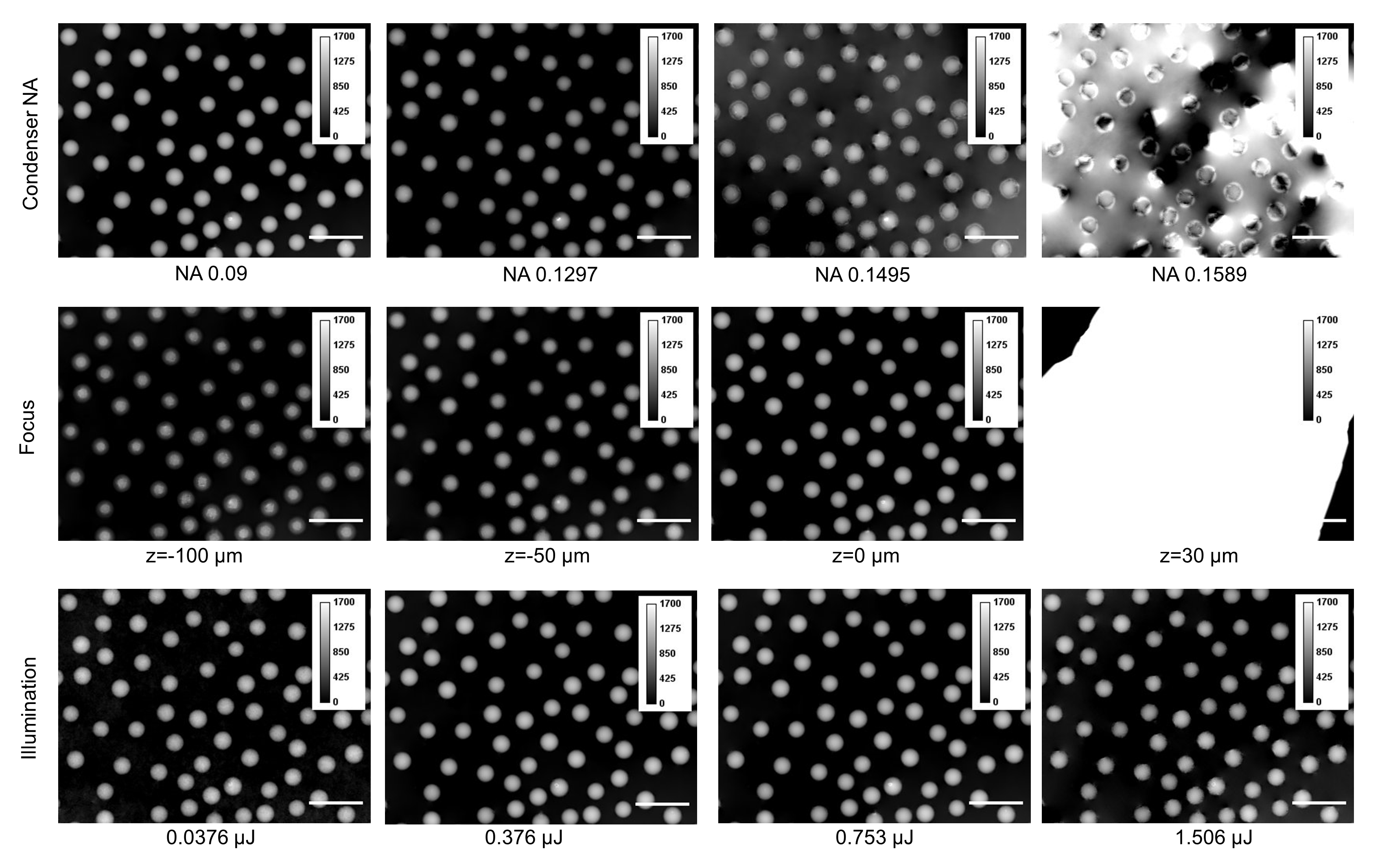

Supplement: Supplemental Figure 1 [file NIHMS1701327-supplement-Supplemental_Figure_1.tif]

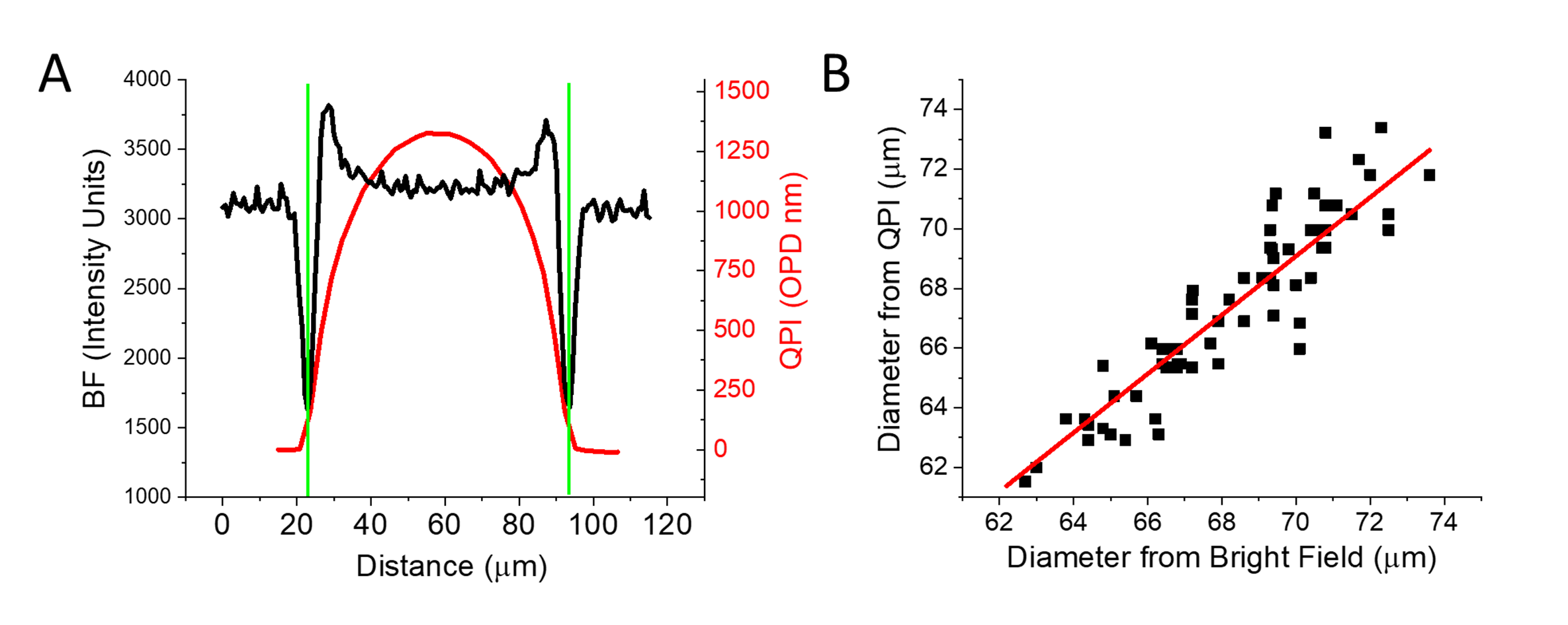

Supplement: Supplemental Figure 3 [file NIHMS1701327-supplement-Supplemental_Figure_3.tif]

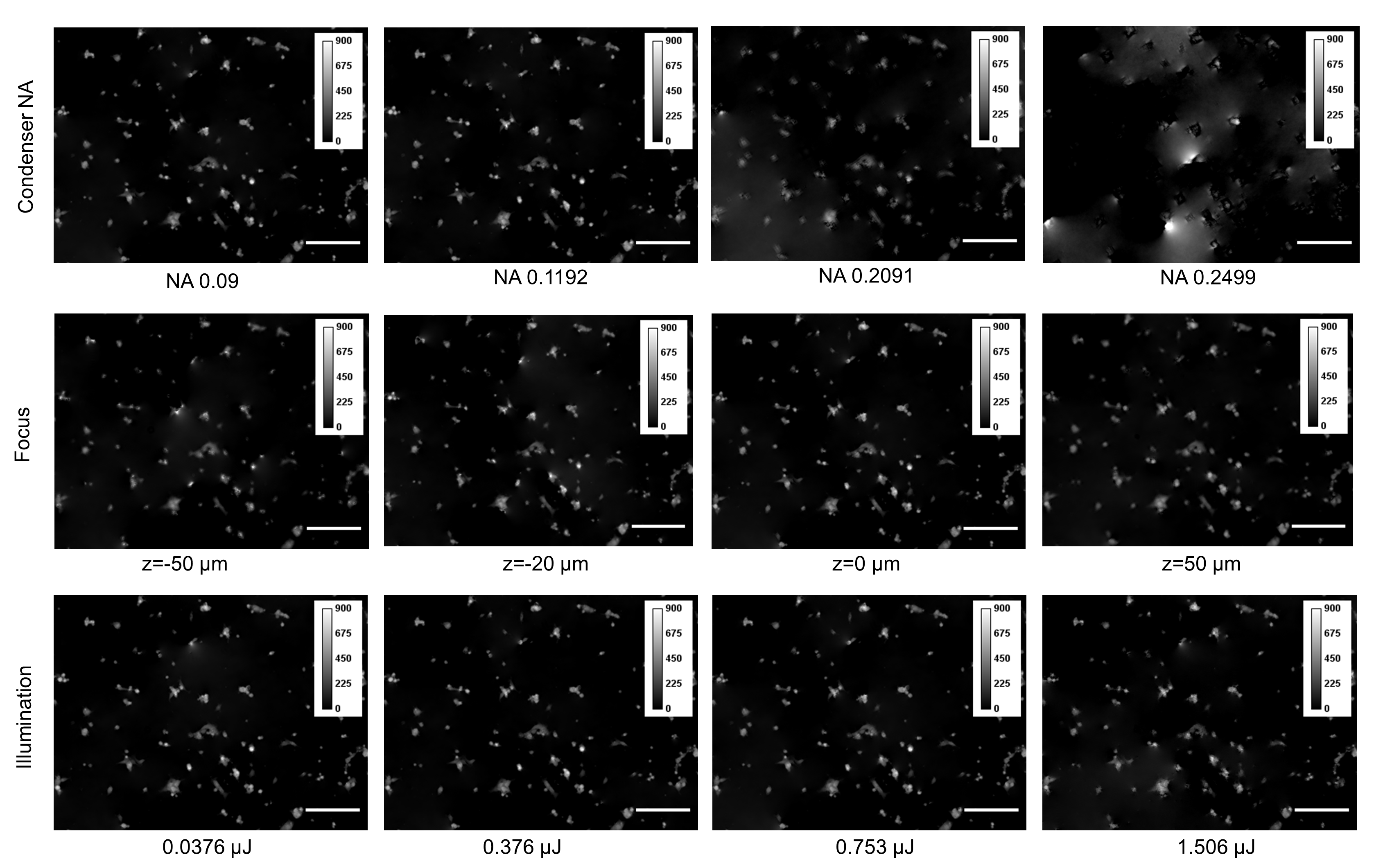

Supplement: Supplemental Figure 2 [file NIHMS1701327-supplement-Supplemental_Figure_2.tif]
