# Supplementary material for: Practical application of microsphere samples for benchmarking a quantitative phase imaging system
Source: Cytometry A. Author manuscript; Available in PMC 2022 Oct 1. (PMC8195315; doi:10.1002/cyto.a.24291)
Supplement: Supplemental Table 1 [file NIHMS1701327-supplement-Supplemental_Table_1.docx]

**Supplemental Table 1: Combinations of microspheres embedded into various media**

| Sample # | Microsphere Material | Microsphere diameter | Microsphere refractive index | Embedding media | Medium refractive index | Calculated *Δn* | Calculated *OPD* (nm) at median diameter |
| --- | --- | --- | --- | --- | --- | --- | --- |
| 1 | Sephacryl | 25 - 75 | 1.358 | DI water | 1.332 | 0.026 | 1300 |
| 2 | Sephacryl | 25 - 75 | 1.358 | Mineral Oil | 1.468 | -0.11 | -5500 |
| 3 | Sephacryl | 25 - 75 | 1.358 | Eukitt | 1.51 | -0.152 | -7600 |
| 4 | Sephacryl | 25 - 75 | 1.358 | MeltMount | 1.539 | -0.181 | -9050 |
| 5 | Sephacryl | 25 - 75 | 1.358 | MeltMount | 1.582 | -0.224 | -11200 |
| 6 | Polyacrylamide | < 50 | 1.36 | DI water | 1.332 | 0.028 | 896 |
| 7 | Polyacrylamide | < 50 | 1.36 | Mineral Oil | 1.468 | -0.108 | -3456 |
| 8 | Polyacrylamide | < 50 | 1.36 | Eukitt | 1.51 | -0.15 | -4800 |
| 9 | Polyacrylamide | < 50 | 1.36 | MeltMount | 1.539 | -0.179 | -5728 |
| 10 | Polyacrylamide | < 50 | 1.36 | MeltMount | 1.582 | -0.222 | -7104 |
| 11 | Silica | 6 | 1.42 | DI water | 1.332 | 0.088 | 528 |
| 12 | Silica | 6 | 1.42 | Mineral Oil | 1.468 | -0.048 | -288 |
| 13 | Silica | 6 | 1.42 | Eukitt | 1.51 | -0.09 | -540 |
| 14 | Silica | 6 | 1.42 | MeltMount | 1.539 | -0.119 | -714 |
| 15 | Silica | 6 | 1.42 | MeltMount | 1.582 | -0.162 | -972 |
| 16 | PMMA | 62 - 75 | 1.484 | DI water | 1.332 | 0.152 | 10032 |
| 17 | PMMA | 62 - 75 | 1.484 | Mineral Oil | 1.468 | 0.016 | 1056 |
| 18 | PMMA | 62 - 75 | 1.484 | Eukitt | 1.51 | -0.026 | -1716 |
| 19 | PMMA | 62 - 75 | 1.484 | MeltMount | 1.539 | -0.055 | -3630 |
| 20 | PMMA | 62 - 75 | 1.484 | MeltMount | 1.582 | -0.098 | -6468 |
| 21 | PS | 6 | 1.59 | DI water | 1.332 | 0.258 | 1548 |
| 22 | PS | 6 | 1.59 | Mineral Oil | 1.468 | 0.122 | 732 |
| 23 | PS | 6 | 1.59 | Eukitt | 1.51 | 0.08 | 480 |
| 24 | PS | 6 | 1.59 | MeltMount | 1.539 | 0.051 | 306 |
| 25 | PS | 6 | 1.59 | MeltMount | 1.582 | 0.008 | 48 |
| 26 | PS | 10 | 1.59 | DI water | 1.332 | 0.258 | 2580 |
| 27 | PS | 10 | 1.59 | Mineral Oil | 1.468 | 0.122 | 1220 |
| 28 | PS | 10 | 1.59 | Eukitt | 1.51 | 0.08 | 800 |
| 29 | PS | 10 | 1.59 | MeltMount | 1.539 | 0.051 | 510 |
| 30 | PS | 10 | 1.59 | MeltMount | 1.582 | 0.008 | 80 |
| 31 | PS | 80 | 1.59 | DI water | 1.333 | 0.257 | 20560 |
| 32 | PS | 80 | 1.59 | Mineral Oil | 1.467 | 0.123 | 9840 |
| 33 | PS | 80 | 1.59 | Eukitt | 1.515 | 0.075 | 6000 |
| 34 | PS | 80 | 1.59 | MeltMount | 1.539 | 0.051 | 4080 |
| 35 | PS | 80 | 1.59 | MeltMount | 1.582 | 0.008 | 640 |
|  |  |  |  |  |  |  |  |

TABLE S1: The selection criteria for the various microsphere/media combinations is that the calculated *Δn* value should be small (*Δn* < 0.05) and the expected *OPD* value should be < 1500 nm to best mimic cellular optical properties. The calculated refractive index change is *Δn* = microsphere refractive index – media refractive index. The calculated *OPD* is the maximum *OPD* at the average reported microsphere diameter (d) according to the equation *OPD* = *Δn⋅D*. The microsphere diameter reported is the manufacturer reported diameter range. When there is a large range of microsphere diameters, such as when the manufacturer specifies only a maximum diameter, image analysis is challenging, specifically due to the absence of a single focal plane and also additional observations of particle debris. The microsphere refractive index is the manufacturer reported value for the PS microspheres, literature reported values for silica, PMMA and Sephacryl [11, 17] and measured using a Phasics SID4BIO camera for polyacrylamide microspheres at 590 nm. It is important to note that the low refractive index microspheres reported here, sephacryl and polyacrylamide, are porous polymer materials and the refractive index of the microsphere will be affected by the type of media in which these microspheres are embedded [17].It should also be noted that we did not measure and evaluate all microsphere/media combinations, such as those combinations that were predicted to have a very large *OPD* (*OPD* > 2000 nm) or refractive index (*Δn* > 0.05) value. It also should be noted that measured refractive index values, for example with silica microspheres, can substantially differ from product specifications usually for smaller sized microspheres [11]. The medium refractive index values are obtained from the literature for distilled water [20] and manufacturer specifications for all others. Six microsphere/media combinations met the selection criteria (colored in green). Because PS at 6 µm and 10 µm are similar in diameter, we selected PS at 6 µm diameter and only further investigated 5 microsphere/media combinations. Several microsphere/media combinations had *OPD* values comparable to cells but slightly higher Δn values than desirable (colored yellow). These combinations were not evaluated further here but may also be suitable for phase calibration materials. For example, for applications taken exclusively at higher magnifications (> 40X) the larger Δn values may not be problematic for QPI software phase reconstruction. While we haven’t reported further on negative microsphere/media results there are two notable examples. First, silica microspheres have an intermediate refractive index value (between water and polystyrene microspheres) that still has a large refractive index change (*Δn* > 0.05) when used in water, but has a negative shift when used in high refractive index media such as mineral oil or solid MeltMount media. In this case, the *Δn* and *OPD* values are low and attenuated; however, they are a negative shift and we wanted to avoid an inverted *OPD* image with media having a larger refractive index than the embedded microsphere. Second, a chemical incompatibility was observed using the PMMA microspheres and Eukitt mounting material. In this case, it appeared that the PMMA microspheres were dissolved by the Eukitt embedding solution.
