# Supplementary material for: Practical application of microsphere samples for benchmarking a quantitative phase imaging system
Source: Cytometry A. Author manuscript; Available in PMC 2022 Oct 1. (PMC8195315; doi:10.1002/cyto.a.24291)
Supplement: Supplemental Table 2 [file NIHMS1701327-supplement-Supplemental_Table_2.docx]

**Supplemental Table 2: Fit for purpose evaluation checklist**

|  | Microspheres in aqueous media (water) | Microspheres in non-aqueous media (mineral oil) | Microspheres in solid embedded media |
| --- | --- | --- | --- |
| Ease of fabrication | ✔ | ✔ | ✖ |
| Ease of implementation | ✔ | ✔ | ✔ |
| Multi-day stability | ✖ | ✔ | ✔ |
| Compatible w/ multi-well plate | ✔ | ✔ | ✖ |
| Compatible with slide holder | ✖ | ✖ | ✔ |
| *OPD* ≈ Adherent cells | ✖ | ✔ | ✔ |
| *OPD* ≈ Nonadherent cells | ✔ | ✔ | ✔ |
| Homogeneous microsphere size distribution | ✖ | ✔ | ✔ |

TABLE S2: The fit-for-purpose evaluation checklist is designed to help assess with the design of calibration material format. It lists several experimental needs and compares with other microsphere formats to guide the user to what format is most suitable and what features are compromised between formats. To summarize, microspheres in aqueous media are the easiest to create and use, however, microsphere compatible with QPI based measurements in water are generally poorly characterized in refractive index and size and are heterogeneous in diameter distribution. Microspheres used that are compatible with non-aqueous media, such as mineral oil, generally can be obtained with homogenous size distributions. Both types of microspheres in liquid media are compatible in well-plate formats identical to cell culture condition format. Microspheres embedded in solid media are generally limited to a microscope slide format; however, the change in refractive index can be better fine-tuned to match the *OPD* level of both adherent and non-adherent cell signal levels. Using a Meltmount in a multi-well plate would require heating the well plate to the Meltmount melting point and then applying the Meltmount to the tissue culture plate. This process would be a problem if there are cell samples in any of the wells in the well plate. Additionally, information from the manufacturer, Cargille, states Meltmount is not compatible with tissue culture polystyrene. For this reason, we recommend PMMA microspheres and mineral oil for well plate formats.
