# Supplementary material for: Practical application of microsphere samples for benchmarking a quantitative phase imaging system
Source: Cytometry A. Author manuscript; Available in PMC 2022 Oct 1. (PMC8195315; doi:10.1002/cyto.a.24291)
Supplement: Supplemental Table 3 [file NIHMS1701327-supplement-Supplemental_Table_3.docx]

Supplemental Table 3

| **Mineral Oil** | **Manufacturer Certificate** | **Measured (n=3)** | |
| --- | --- | --- | --- |
| **Lot** | **Refractive Index *n*** | **Average Refractive Index *n*** | **Standard Deviation** |
| BCBL0289V | 1.471 | 1.470 | 0.0001000 |
| BCCB9373 | 1.470 | 1.469 | 0.0001000 |
| BCCD1059 | 1.467 | 1.466 | 0.0001528 |

Supplemental Table 3: Comparison of manufacturer specified refractive index and measured refractive index of mineral oil. Three different lots of mineral oil (BioUltra, Sigma-Aldrich, St. Louis, MO) were purchased. The refractive index was independently confirmed using a PAL-RI refractometer (Atago, Tokyo, Japan) calibrated to the refractive index of water. Measured refractive index were in close agreement with manufacturer provided specifications. Average refractive index and standard deviation determined from three independent measurements.
