# Supplementary material for: Practical application of microsphere samples for benchmarking a quantitative phase imaging system
Source: Cytometry A. Author manuscript; Available in PMC 2022 Oct 1. (PMC8195315; doi:10.1002/cyto.a.24291)
Supplement: Supplemental Table 4 [file NIHMS1701327-supplement-Supplemental_Table_4.docx]

Supplemental Table 4

| **PMMA Lot** | **Mineral Oil Lot** | **Average *Δn*** | **Standard Deviation** | **Measured PMMA refractive index *n*** | **Number of microspheres** | **Average PMMA refractive index *n*** |
| --- | --- | --- | --- | --- | --- | --- |
| U99-1046 | BCBL0289V | 0.0154 | 0.000899 | 1.486 | 114 | 1.486 |
| U99-1046 | BCCB9373 | 0.0188 | 0.000828 | 1.486 | 169 |  |
| U99-1046 | BCCD1059 | 0.0164 | 0.000642 | 1.486 | 205 |  |
| 180420-102-1-2-1 | BCBL0289V | 0.0184 | 0.000977 | 1.489 | 235 | 1.490 |
| 180420-102-1-2-1 | BCCB9373 | 0.0204 | 0.001334 | 1.487 | 52 |  |
| 180420-102-1-2-1 | BCCD1059 | 0.0219 | 0.001021 | 1.492 | 145 |  |

Supplemental Table 4: Evaluation of different lot combinations of PMMA and mineral oil. Using two lots of PMMA microspheres (Cospheric, Santa Barbara, CA) and three lots of mineral oil (BioUltra, Sigma-Aldrich, St. Louis, MO), six different combinations of reference material preparations were made. Phase images were acquired and at least seven images and 50 microspheres were analyzed for each preparation. Average PMMA refractive index was calculated from the measured average with the three different mineral oil lots.
