# Supplementary material for: Practical application of microsphere samples for benchmarking a quantitative phase imaging system
Source: Cytometry A. Author manuscript; Available in PMC 2022 Oct 1. (PMC8195315; doi:10.1002/cyto.a.24291)
Supplement: Supplemental Table 5 [file NIHMS1701327-supplement-Supplemental_Table_5.docx]

Supplemental Table 5

| **Preparation** | **Average *Δn*** | **Standard Deviation** | **Measured PMMA refractive index *n*** | **Number of microspheres** | **Average PMMA refractive index *n*** |
| --- | --- | --- | --- | --- | --- |
| 1 | 0.0154 | 0.000899 | 1.486 | 114 | 1.487 ± 0.000420 |
| 2 | 0.0152 | 0.000669 | 1.486 | 60 |  |
| 3 | 0.0162 | 0.000453 | 1.487 | 34 |  |
| 4 | 0.0161 | 0.000612 | 1.487 | 31 |  |
| 5 | 0.0160 | 0.000316 | 1.487 | 46 |  |

Supplemental Table 5: Reference material preparation variation. For the same lot of PMMA microspheres and mineral oil, five independent preparations were made and analyzed using QPI. The average induced *Δn* and PMMA refractive index were calculated across all microspheres in the preparation. The average PMMA refractive index and standard deviation was calculated across the five preparations.
